# Quality of Life before and after Sleeve Gastrectomy in Lebanese Population

**DOI:** 10.1155/2019/1952538

**Published:** 2019-08-06

**Authors:** Marwan Alkassis, Fady Gh Haddad, Joseph Gharios, Roger Noun, Ghassan Chakhtoura

**Affiliations:** ^1^General Surgery Department, Saint Joseph University, Beirut, Lebanon; ^2^Hematology-Oncology Department, Saint Joseph University, Beirut, Lebanon

## Abstract

**Introduction:**

Obesity is increasing worldwide and in Lebanon with a negative impact on the quality of life. The primary objective of this study is to measure the quality of life in obese subjects before and after bariatric surgery, depending on age, sex, and degree of weight loss. A secondary objective is to determine the impact of bariatric surgery on comorbidities associated with obesity.

**Materials and methods:**

Patients undergoing laparoscopic sleeve gastrectomy for BMI ≥ 30 kg/m^2^ between August 2016 and April 2017 were included. Participants completed the Moorehead-Ardelt Quality of Life Questionnaire II (MA II) prior to operation and one year after. Statistical analysis was carried out using SPSS statistics version 20.0.

**Results:**

75 patients participated in the study. The majority were women (75%), and the mean age was 36.3 years. The mean weight loss was 36.57 kg (16–76). Initially, the total MA II score was −0.33 ± 0.93. Postoperatively, it increased to 1.68 ± 0.62 (*p* ≤ 0.001). All MA II parameters improved after surgery (*p* ≤ 0.001), but this improvement was independent of age and sex. Improvement in self-esteem, physical activity, work performance, and sexual pleasure was influenced by the degree of weight loss (*p* ≤ 0.001). All comorbidities associated with obesity regressed significantly after sleeve gastrectomy (*p* < 0.05) with the exception of gastroesophageal reflux and varicose veins of the lower limbs.

**Conclusion:**

Sleeve gastrectomy improves quality of life and allows reduction of comorbidities.

## 1. Introduction

Obesity has more than doubled in the last 30 years [1] and represents a major health problem because of its economic consequences and its association with several comorbidities. Obesity increases the prevalence of high blood pressure, type 2 diabetes, type 1 diabetes, heart disease, stroke, cancer, and mental illness [2]. All these comorbidities, in addition to the discrimination and stigmatization of society, alter the quality of life of the obese patients in all its aspects [3]. Several therapeutic modalities exist to reduce the excess weight; however, the surgical techniques remain the most effective [4]. The two most practiced techniques throughout the world are gastric bypass Roux-en-Y and sleeve gastrectomy [5]. They allow a significant reduction in weight and a reduction in type 2 diabetes, high blood pressure and mortality from cancer, stroke, and cardiovascular disease [6]. Quality of life can be assessed by the Moorehead-Ardelt Quality of Life Questionnaire II or the 36-Item Short Form Survey. Some studies have noted an improvement in the quality of life after bariatric surgery [7–9], while others have noted an absence of improvement or a decline [10].

The rate of obesity in Middle Eastern countries is increasing alarmingly and Lebanon is no exception [11]. According to a study published in 2016, 53.5% of the Lebanese population is overweight and 18.16% is obese [12]. In parallel with the increase in the rate of obesity, we note an increase in the use of bariatric surgery in the Middle East [13]. The studies carried out in Lebanon concerning sleeve gastrectomy are few and there is not yet any nationwide study comparing the quality of life before and after sleeve gastrectomy. Hence, the originality of this prospective study whose objective is to compare the quality of life of patients before, and one year after sleeve gastrectomy, is using the Moorehead-Ardelt Quality of Life Questionnaire II (MA II) as a scale of measurement of the quality of life.

## 2. Materials and Methods

### 2.1. Study Design

This is a prospective study in obese subjects (BMI ≥ 30 kg/m^2^) operated of laparoscopic sleeve gastrectomy between August 2016 and April 2017 in Hotel-Dieu de France Hospital, a tertiary center in Lebanon. The inclusion criteria were as follows:

- BMI ≥ 35 kg/m^2^, regardless of the presence or absence of comorbidities
- BMI ≥ 30 kg/m^2^ with comorbidities
- Age between 18 and 65 years
- No previous bariatric or gastric surgery

The study was approved by the Ethics Committee of Hotel-Dieu de France Hospital and the Faculty of Medicine of Saint Joseph University. The purpose and the course of the study were communicated to all participants who, after understanding the information, signed an informed consent.

### 2.2. Data Collection

The study was conducted in two stages: preoperative and postoperative. The preoperative stage involved the collection of patient-specific data such as age, occupation, social status, associated comorbidities, and medical and surgical history. This step also involved a complete clinical examination, a weight and height measurement (to calculate BMI and the mean weight loss), and a preoperative quality of life questionnaire. The participants were operated of laparoscopic sleeve gastrectomy. The postoperative stage took place one year after the date of the operation, during which the subjects were contacted by telephone or by e-mail, and the same information collected preoperatively was collected postoperatively, including the quality of life questionnaire.

### 2.3. Quality of Life Questionnaire

Quality of life was assessed by the Moorehead-Ardelt Quality of Life Questionnaire II (MA II). This validated questionnaire for the measurement of the quality of life in obese subjects studies 6 main parameters of the quality of life: self-esteem, physical activity, social life, work performance, pleasure obtained during the sexual relation, and the food approach. Each section has 10 boxes rated from “1” to “10” corresponding to the answer that the patient finds most appropriate, with “1” being the most pejorative answer while “10” is the best possible score. Each answer corresponds to a coding system which varies from “−0.5” to “+0.5”.

The patient is free not to answer one or more of the questions, and for any missing answer, a score of “0” has been assigned. Once the questionnaire is completed, a final grade is obtained, and it varies between “−3” and “+3”. The final interpretation is done through intervals: the quality of life is very poor when the final score is between “−3” and “−2.1,” poor when it is between “−2” and “−1.1,” fair when it is between “−1” and “+1,” good when it is between “+1.1” and “+2,” and very good when it is between “2.1” and “+3”. All patients received a questionnaire in English and an oral explanation for subjects with difficulty understanding English. Once the instructions were well assimilated, the responses were noted personally by the patients. Since the postoperative phase was conducted by telephone or e-mail, the 6 questions were narrated by the same “interviewer” for all patients. For patients contacted by e-mail, a copy of the questionnaire was attached to the instructions.

### 2.4. Statistical Analysis

The data collected were then analyzed. Chi-square or Fisher test was used in the analysis of two categorical variables, while the paired *t*-test was used in the comparison of mean between 2 matched/dependent groups, and independent-samples *t*-test was used when comparing a categorical variable and a numerical one between 2 independent groups. In addition, correlations were made between two numerical variables. The significance level was set for *p* value <0.05.

Analyses were performed using the software “SPSS statistics version 20.0”.

## 3. Results

One hundred and six patients completed the initial preoperative questionnaire. 31 patients were lost to follow-up, and a total of 75 patients were included in this study.

The majority of the participants (56) were women (75%). The mean age was 36.3 years (18–62). Mean preoperative weight was 111.25 kg (83–156).

Comorbidity was deemed present when the patient was under medication. Of the participants, 19 (25.3%) patients were hypertensive, 13 (17.3%) were diabetic, and 20 (26.7%) were dyslipidemic. Sleep apnea (AS) was found in 25 (33.3%) patients, defined by the presence of symptoms reported by the patient or by members of his family, such as snoring, daytime sleepiness, morning headache, and the observation of sleep apnea by those around him. Polysomnographic studies were not performed. 27 (36%) had clinical gastroesophageal reflux disease (GERD), and 11 (14.7%) had varicose veins in the lower extremities. 25 women (44%) in the study had menstrual irregularities. 38 (50.7%) patients had chronic low back pain, 32 (42.7%) had chronic gonalgia, and 9 (12%) had chronic coxalgia. No patient had a history of myocardial infarction (Table 1).

Based on the responses of participants to the MA II questionnaire, the total preoperative quality of life score was −0.33 ± 0.93. Only one patient had a very poor quality of life, 19 (25.3%) patients had a poor quality of life, 52 (69%) had a fair quality of life, 3 (4%) had a good quality of life, and no participant had a very good quality of life.

A significant reduction in weight (*p* ≤ 0.001) was noted after surgery. The mean postoperative weight was 74.68 kg varying between 56 and 119 kg. The mean postoperative weight loss was 36.57 kg varying between 16 and 76 kg. A significant decrease in comorbidities was observed. Hypertension resolved in 11 patients of 19 (57.9%), diabetes resolved in 11 patients of 13 (84.61%), and dyslipidemia in 13 patients of 20 (65%). Of the initial 25 patients, only 2 (2.7%) continued to suffer from sleep apnea syndrome. There was complete resolution of menstrual irregularities in 16 patients (64%). A significant decrease in joint pain was also noted. However, the reduction in GERD and lower extremity varicose veins was not significant with the persistence of these disorders in 18 (24%) and 7 (9.3%) participants, respectively (Table 2).

**Figure 1:**
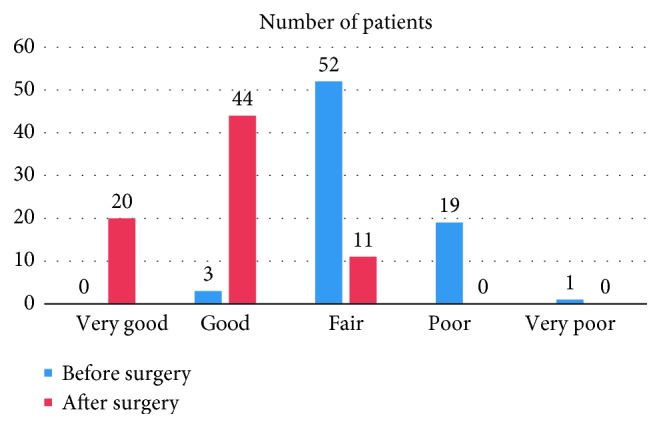
Analysis of changes in the quality of life before and after surgery according to MA II.

There was a significant improvement in the total MA II score with a value of 1.68 ± 0.62 (*p* ≤ 0.001).  Table 3 exposes a more detailed analysis of the different MA II parameters showing a significant improvement in all scales (*p* ≤ 0.001). In addition, postoperatively, no patient had a very poor or poor quality of life, whereas 11 (15%), 44 (58.7%), and 20 (26.7%) patients had a fair, good, and very good quality of life, respectively (Figure 1).

We also looked if the improvement in each MA II parameter differed before and after surgery according to age groups (<30 vs. ≥30 years), gender (men vs. women), and percentage of weight loss (<30% vs. ≥30%).  Table 4 shows that the improvement in MA II parameters was independent of age, gender, and percentage of weight loss, with no statistical difference observed (*p* > 0.05).

## 4. Discussion

The remarkable increase in global obesity rates and comorbidities has made obesity a major health and economic problem. Lebanon is no exception and the current trend is towards an increase in the number of overweight and obese people in Lebanon [12].

Obesity confers a high risk of mortality because of the multiple associated comorbidities [2]. The association between obesity and cardiovascular risk factors has been well demonstrated in multiple studies as well as in this study. Of the 75 participants, 25.3% were hypertensive on treatment, 17.3% were diabetic on treatment, and 26.7% were dyslipidemic on treatment. These numbers are consistent with those of several previous studies [14–17]. Postoperatively, a significant fall in the rate of arterial hypertension (10.7% vs. 25.3%), diabetes (2.7% vs. 17.3%), and lipid disorders (9.3% vs. 26.7%) was noted.

In this study, the effect of weight loss by sleeve gastrectomy on other comorbidities, including sleep apnea, menstrual bleeding, arthralgia, gastroesophageal reflux disease (GERD), and varicose veins, was also studied There was a significant decrease in sleep apnea, menstrual irregularities, and joint pain. However, the decrease in GERD was not significant. This can be explained by the fact that bariatric surgery and especially sleeve gastrectomy can cause or exacerbate GERD, as reported by Chiu et al. [18]. In addition, improvement in varicose veins of the lower extremities was not significant because obesity in this pathology is only one factor among other predominant.

Obesity, through its comorbidities and the stigmatization of obese subjects, alters the quality of life [10]. This assumes that decreasing weight improves the quality of life. The objective of this study was to confirm this hypothesis. In this study, the overall preoperative MA II score was −0.33 ± 0.93; this figure improved significantly (*p* ≤ 0.001) after sleeve gastrectomy to reach 1.68 ± 0.62. This result is consistent with that obtained by Janik et al. who used another methodology comparing the quality of life according to MA II in obese subjects operated by sleeve gastrectomy or gastric bypass Roux-en-Y and nonoperated obese subjects. He noticed a marked improvement in the overall quality of life in the operated subjects (1.70 ± 0.76 vs. 0.59 ± 1.17, *p* value ≤ 0.01) [19]. Zhang et al. also used the MA II to compare the quality of life between the preoperative and postoperative state in obese subjects operated by sleeve gastrectomy or gastric bypass Roux-en-Y. He concludes a clear improvement in the quality of life postoperatively, without noticing a significant difference between the group operated by sleeve gastrectomy and the group operated by gastric bypass Roux-en-Y [20], a result similar to that of Janik et al. [19]. Other authors have used the SF-36 as a tool for measuring the quality of life and have noticed a significant improvement in the quality of life after bariatric surgery [9, 21, 22].

In our study, a detailed analysis of the quality of life showed a significant improvement in all areas of MA II (*p* ≤ 0.001): self-esteem, physical activity, social life, work performance, sexual pleasure, and the food approach. Indeed, during the interrogation one year after the surgery, the majority of the participants express a clear increase in self-confidence. Some report that they no longer have difficulty finding clothes suitable for their size in contrast to their preoperative condition when they were often forced to isolate themselves socially. An important element of MA II is the dietary approach. Many patients report that food plays a less important role in their lives after surgery. This may be related to early satiety, epigastric pain, and GERD experienced postoperatively by some patients. Mans et al. noted that sleeve gastrectomy plays a role in reducing hunger and increasing satiety by accelerating gastric emptying, reducing plasma ghrelin concentration, and increasing postprandial serum cholecystokinin and glucagon-like peptide 1 [23]. Our results are not quite consistent with the study by Janik et al. Indeed, he noted a significant improvement in self-esteem, physical activity, social life, and the dietary approach (*p* < 0.01), while, unlike our results, work performance and sexual pleasure improved nonsignificantly (*p* = 0.97 and 0.10, respectively) [19].

The MA II classifies the different scores obtained in qualitative groups. In this study, and preoperatively, a single patient had a very poor quality of life. 19 patients (25.3%) had a poor quality of life, 52 (69.4%) had a fair quality of life, 3 (4%) had a good quality of life, and no participant had a very good quality of life. Postoperatively, no patient had a very poor or poor quality of life, while 11 (14.6%), 44 (58.7%), and 20 (26.7%) patients had fair, good, and very good quality of life, respectively. These results reflect a significant improvement in the quality of life after sleeve gastrectomy.

These results are similar to those of Major et al. who reported preoperatively 1.6% of subjects with very poor quality of life, 21.5% with poor quality of life, 58.4%, 13.8%, and 4.7% of patients with fair, good, and very good quality of life, respectively. Postoperatively, no patient had a very poor quality of life, and most patients had a good and very good quality of life (36.4% and 33.8%, respectively) [9]. The same result was reported by Janik et al. where no patient had a very bad or bad quality of life after sleeve gastrectomy or gastric bypass Roux-en-Y [19].

In his study, Janik et al. called for a better assessment of the role of age, sex, and relational status of the patient in evaluating the quality of life after bariatric surgery [19]. In our study, age and sex were not correlated with the degree of improvement in the quality of life. The study population was divided into 2 groups, those under 30 and those aged 30 and above with the idea that very young subjects opted for bariatric surgery for a rather aesthetic purpose, whereas for older subjects, medical considerations predominate. There was no significant difference (*p* > 0.05) between these 2 groups in the improvement of the 6 parameters of MA II. Similarly, the improvement in the quality of life in all areas was comparable for men and women, and between patients losing less than 30% or 30% and more of body weight.

Some studies have noted an impairment of quality of life after bariatric surgery [10]. This was not found in our study where all patients noted an improvement in their quality of life. It is interesting to note, however, that one patient complained of excess skin mass after weight loss and another patient complained of the excessive degree of weight loss.

Overweight is certainly a big problem in life, but it should not be the scapegoat for all the problems of the obese patient. Any overweight person will tend to put his inability to find work, to find a partner, or even to find a circle of friends on account of his physical appearance. These patients expect major changes in their lives after the surgery, and some will inevitably be disappointed when they discover that their life did not change drastically as hoped; failure to achieve this goal will have a negative impact on their psychological health even when weight loss is achieved. For this reason, it is the role of the surgeon to guide patients in their expectations toward the surgery, focusing primarily on improving the physical health without selling bariatric surgery as a magic stick able to make disappear all the difficulties in daily life.

However, the cost of the treatment, follow-up, or management of short- and long-term complications of the multiple comorbidities is high and exceeds the cost of sleeve gastrectomy. In addition, the medical benefits of bariatric surgery are numerous and proven. Thus, a single surgical procedure resolves a very large number of medical problems and subsequently reduces medical expenses. This encourages governments to take care of these patients for free to reduce comorbidities and subsequently medical expenses. This policy is especially interesting in countries like Lebanon, where the healthcare system is not well developed as the vast majority of people do not have medical coverage.

## 5. Conclusion

This study demonstrated that obese subjects undergoing laparoscopic sleeve gastrectomy had a better quality of life score according to MA II. This improvement is dependent on the degree of weight loss and independent of age and gender. The satisfaction engendered by bariatric surgery seems to come from its improvement in both physical and mental health. However, we should avoid excessive optimism and concentrate on the general aspect without selling a better tomorrow that may never come.

## Figures and Tables

**Table 1:** Characteristics of patients and prevalence of comorbidities associated with obesity before sleeve gastrectomy.

Patients characteristics	Variables	% (*N*)
Gender	Women	74.7 (56)
	Men	25.3 (19)
Comorbidities	Arterial hypertension	25.3 (19)
	Diabetes	17.3 (13)
	Dyslipidemia	26.7 (20)
	Smoking	16.0 (12)
	Sleep apnea	33.3 (25)
	Gastroesophageal reflux	36.0 (27)
	Varicose veins	14.7 (11)
	Menstrual irregularities	33.3 (25)
	Low-back pain	50.7 (38)
	Gonalgia	42.7 (32)
Mean age in years (±SD)	36.3 ± 10.2	
Mean weight in kg (±SD)	111.25 ± 4.1	

**Table 2:** Comparison of comorbidities among patients before and after sleeve gastrectomy.

Comorbidities	Before surgery % (*N*)	After surgery % (*N*)
Arterial hypertension^*∗*^	25.3 (19)	10.7 (8)
Diabetes^*∗∗*^	17.3 (13)	2.7 (2)
Dyslipidemia^*∗∗*^	26.7 (20)	9.3 (7)
Sleep apnea^Δ^	33.3 (25)	2.7 (2)
Gastroesophageal reflux	36.0 (27)	24 (18)
Varicose veins	14.7 (11)	9.3 (7)
Menstrual irregularities^*∗∗*^	33.3 (25)	12 (9)
Low-back pain^Δ^	50.7 (38)	16 (12)
Gonalgia^∆^	42.7 (32)	9.3 (7)
Coxalgia^*∗*^	12 (9)	2.7 (2)

^*∗*^ *p* < 0.05; ^*∗∗*^ *p* < 0.01; ^Δ^ *p* ≤ 0.001.

**Table 3:** Comparison of the score of the various MA II parameters before and after surgery.

MA II parameters	Score	
	Before surgery	After surgery
Self-esteem^Δ^	−0.18 ± 0.26	0.37 ± 0.14
Physical activity^Δ^	−0.27 ± 0.22	0.29 ± 0.20
Social life^Δ^	0.10 ± 0.31	0.39 ± 0.13
Work performance^Δ^	0.13 ± 0.28	0.33 ± 0.14
Sexual pleasure^Δ^	−0.004 ± 0.22	0.21 ± 0.19
Food approach^Δ^	−0.11 ± 0.28	0.10 ± 0.24
Total score^Δ^	−0.33 ± 0.93	1.68 ± 0.62

^*∗*^ *p* < 0.05; ^*∗∗*^ *p* < 0.01; ^Δ^ *p* ≤ 0.001.

**Table 4:** Variation of MA II parameters according to age, gender, and percentage of weight loss.

MA II parameters	Age		Gender		Percentage of weight loss	
	<30 years	≥30 years	Men	Women	<30%	≥30%
Self-esteem	0.48 ± 0.29	0.57 ± 0.29	0.54 ± 0.22	0.54 ± 0.31	0.50 ± 0.29	0.56 ± 0.29
Physical activity	0.51 ± 0.29	0.57 ± 0.26	0.57 ± 0.16	0.55 ± 0.29	0.51 ± 0.31	0.57 ± 0.25
Social life	0.23 ± 0.31	0.30 ± 0.31	0.28 ± 0.32	0.28 ± 0.31	0.35 ± 0.35	0.26 ± 0.29
Work performance	0.21 ± 0.31	0.20 ± 0.33	0.19 ± 0.27	0.20 ± 0.34	0.22 ± 0.36	0.19 ± 0.31
Sexual pleasure	0.17 ± 0.27	0.24 ± 0.27	0.21 ± 0.28	0.22 ± 0.27	0.22 ± 0.29	0.22 ± 0.27
Food approach	0.21 ± 0.34	0.21 ± 0.34	0.11 ± 0.32	0.24 ± 0.33	0.30 ± 0.33	0.18 ± 0.33

^*∗*^ *p* < 0.05; ^*∗∗*^ *p* < 0.01; ^Δ^ *p* ≤ 0.001.

## Data Availability

The data used to support the findings of this study are available from the corresponding author upon request.

## Supplementary Materials

Supplementary Materials The tables representing the data used to support the findings of this study are included within the supplementary information file.
